# Electrochemical behavior of Mg electrode in sodium salt electrolyte system

**DOI:** 10.3389/fchem.2022.992400

**Published:** 2022-09-09

**Authors:** Yu Zhang, Qingguang Zhu, Chang Su, Chao Li

**Affiliations:** ^1^ College of Chemistry and Chemical Engineering, Xinyang Normal University, Xinyang, China; ^2^ Xinyang Key Laboratory of Low-Carbon Energy Materials, Xinyang Normal University, Xinyang, China

**Keywords:** AZ31B Mg alloy, composite electrolyte, Na_2_SiO_3_, electrochemical performance, corrosion behavior

## Abstract

A suitable electrolyte is crucial to enhancing the electrochemical performance of magnesium (Mg) batteries. Here, the influence of Na_2_SiO_3_ on the electrochemical behavior of AZ31B Mg alloy in the Na_2_SO_4_-NaNO_3_ composite electrolyte was investigated. The results revealed that the activation potential of the AZ31B Mg alloy first represented a negative shift and then a positive shift with the increase in Na_2_SiO_3_. The most negative activation potential (−1.51 V) and the lowest polarization (−3.20 V) were found when 6 mM of Na_2_SiO_3_ was added; no discharge hysteresis was observed, and the polarization resistance value (*R*_1_) was 3,806 Ω. After 24 h immersion in the composite electrolyte with Na_2_SiO_3_, more and wider cracks appeared on the alloy surface, where a thick, dense film was formed, showing excellent discharge performance and corrosion resistance.

## Introduction

In view of the limited nature and high price of lithium resources, the research on electrochemical energy storage devices, such as non-lithium batteries (Zhang J. L. et al., 2021; Liu et al., 2021; Wan et al., 2022) and supercapacitors (Zhang Y. et al., 2021; Wei et al., 2021) is increasing. Magnesium (Mg) holds a promising application in anode materials for the first-generation Mg battery owing to its abundance, small density (1.74 g cm^−3^), low cost, and excellent electrical conductivity (Bertasi et al., 2016; Yang et al., 2022). In addition, located on the diagonal of the periodic table, Mg and Li share many similar chemical properties. The standard electrode potential of metallic Mg is −2.36 V (vs. SHE), allowing the formation of large open-circuit voltage and working voltage (Deng et al., 2019; Maddegalla et al., 2021). Mg has an electrochemical equivalent of 0.454 g Ah^−1^ and a theoretical specific capacity up to 2,202 mAh g^−1^, enabling it to be applied to long-time discharge (Kékedy-Nagy et al., 2021). However, shortcomings also remain unresolved, such as the lessened battery storage capacity after discharge, voltage hysteresis, severe inferior corrosion resistance of the electrode, and poor current efficiency, forming the main obstructors of the commercial availability of Mg battery (Shao et al., 2015; Horia et al., 2022; Zhang et al., 2022). In the Mg battery, “voltage lag” is the essence of the passivation of the Mg electrode in the electrolyte. Passivation film hinders the reaction during battery discharge, and its breakdown promotes a smooth reaction. Therefore, a Mg battery is required for the normal voltage output over a period of time. The “hysteresis” in the Mg battery is mainly relevant to the coverage degree of surface passivation film and the rate and relaxation time of film breakdown (Gong et al., 2022; Wei et al., 2022).

Recently, employing a suitable additive into electrolytes has been proved to be simple and effective in changing the surface membrane structure of the Mg electrode, thus reducing voltage lag (Li et al., 2021). The presence of both 0.005 M EDTA-ZnNa_2_ and 0.01 M C_6_H_11_NaO_7_ has been demonstrated to remarkably inhibit corrosion and improve the performance of Mg battery for pure Mg in 3.5 wt% NaCl electrolyte (Qu et al., 2022). Zhao et al. (2016) investigated the discharge performance of an oxyanion corrosion inhibitor (Li_2_CrO_4_) as an electrolyte additive in 3.5 wt% NaCl electrolyte for Mg-air battery, verifying enormously reduced corrosion current density of AZ31B Mg alloys in the presence of 0.1 wt% Li_2_CrO_4_, which is beneficial to the intermittent discharge performance of the Mg-air battery.

Sodium metasilicate (Na_2_SiO_3_) is relatively cheap, environment-friendly, and non-toxic, often used as an anionic corrosion inhibitor (Kong et al., 2022). This thesis mainly explored the electrochemical performance and corrosion behavior of AZ31B Mg alloy in Na_2_SO_4_-NaNO_3_ and Na_2_SiO_3_ composite electrolyte to pick out the matching electrolyte, thus ensuring the activation of the electrode and inhibiting the hydrogen evolution-induced self-corrosion.

## Experiment

### Chemicals and materials

In this study, Na_2_SO_4_, NaNO_3_, and Na_2_SiO_3_ of analytical grade (≥99%) were utilized. The electrolyte was obtained by a mixture of 2 M Na_2_SO_4_ and 2 M NaNO_3_ (volume ratio = 1:9), and the Na_2_SiO_3_ concentration ranged from 0.2 to 1.0 mM.

According to our previous work (Xu et al., 2017), the AZ31B Mg alloy (3.0 wt% Al, 1.0 wt% Zn, 0.2 wt% Mn, and 96.8 wt% Mg) was purchased from Wuxi Xinbiao Metal Material Co. Ltd. in China. The Mg alloy was sheared into an appropriate size of 1 × 1 cm with a thickness of 0.6 cm, then embedded with electric conductive copper wire, and encapsulated with epoxy resin in quick succession. Subsequently, these prepared samples were used as the working electrode for the electrochemical analysis.

### Electrochemical characterization

The electrochemical characterization was performed on an electrochemical measurement system (CHI660E, China) using a standard three-electrode system comprising the graphite rod as the counter electrode, the saturated calomel electrode as the reference electrode, and the AZ31B Mg alloy as the working electrode.

The influence of Na_2_SiO_3_ additive on the electrochemical behavior of AZ31B Mg alloy electrode in the Na_2_SO_4_-NaNO_3_ composite electrolyte was investigated by the linear sweep voltammetry (LSV) at a sweep rate of 1 mV s^−1^. The discharge curves were explored at a discharge current density of 3 mA cm^−2^. Moreover, the electrochemical impedance spectroscopy (EIS) was conducted at open circuit potentials in the range of 10^–2^∼10^5^ Hz with an amplitude of 5 mV.

### Surface morphology analysis

The effect of the Na_2_SiO_3_ additive on the morphology of the corrosive film layer on the Mg alloy electrode surface immersed into the Na_2_SO_4_-NaNO_3_ composite electrolyte with or without Na_2_SiO_3_ (0.6 mM) for 24 h was revealed by the scanning electron microscopy (SEM, HITACHI S 4800) at an operating voltage of 15 kV.

## Results and discussion

**FIGURE 1:**
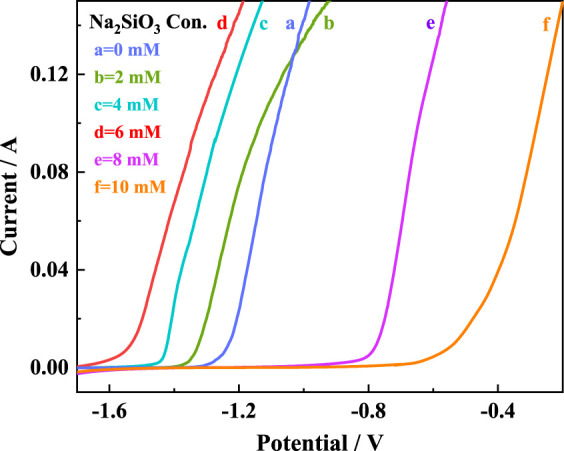
LSV curves of AZ31B Mg alloy electrodes in Na_2_SO_4_-NaNO_3_ composite electrolyte with different concentrations of Na_2_SiO_3_.

The AZ31B Mg alloy electrodes were soaked in the Na_2_SO_4_-NaNO_3_ composite electrolytes of different Na_2_SiO_3_ concentrations ( $C_{{\text{Na}}_{2}{\text{SiO}}_{3}}$ ) for 24 h. Then, the LSV curves of the electrodes were measured at a sweep rate of 1 mV s^−1^, and the results are shown in Figure 1. The inflection point on the curve is generally referred as the activation electrode potential (*E*_act_). Generally, negative values of *E*_act_ imply a low self-corrosion rate and a high discharge activity, under which the Mg alloy is strongly corrosion-resistant (Wang et al., 2014). Based on the curves, Na_2_SiO_3_ addition led to negative shifts in *E*_act_, indicating the ability of Na_2_SiO_3_ to improve the discharge behavior of the Mg alloy electrode in the composite electrolyte. With the increase in $C_{{\text{Na}}_{2}{\text{SiO}}_{3}}$ , *E*_act_ values shifted negative first and then positive. *E*_act_ reached to lowest point (−1.51 V) when $C_{{\text{Na}}_{2}{\text{SiO}}_{3}}$ was 6 mM, 1.1 V poorer than the original value without Na_2_SiO_3_ additive. A plausible reason was that the Na_2_SiO_3_ addition affected the ionization equilibrium by accelerating the release of Mg^2+^ ions from the Mg alloy, consequently improving the ionic conductivity and the mass transfer of Mg^2+^ ions (Zhang et al., 2022). The shift of *E*_act_ values might also be closely related to the microstructural change of the Mg alloy in the composite electrolyte added with Na_2_SiO_3_, as verified by the SEM images (vide infra).

**FIGURE 2:**
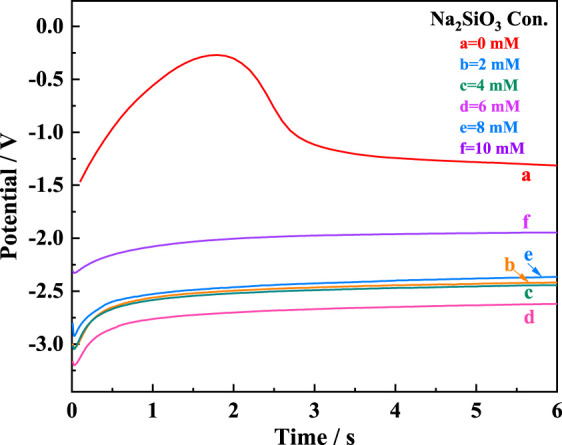
Discharge curves of AZ31B Mg alloy electrode in the Na_2_SO_4_-NaNO_3_ composite electrolyte with different $C_{{\text{Na}}_{2}{\text{SiO}}_{3}}$ at a current density of 3 mA cm^−2^.

Figure 2 illustrates the discharge curves of AZ31B Mg alloy electrodes in the composite electrolytes with varying $C_{{\text{Na}}_{2}{\text{SiO}}_{3}}$ at a discharge current density of 3 mA cm^−2^. In the absence of Na_2_SiO_3_, the discharge potential reached its maximum rapidly at the initial discharge stage and then slowly recovered to a stable state, which was ascribed to the activation process. This period lasted for 2.7 s and was attributed to the “hysteresis time.” Surprisingly, the “hysteresis time” disappeared after the addition of Na_2_SiO_3_, and the discharge curves were rapidly stabilized. Na_2_SiO_3_, as a type of surfactant, loosened the passive film on the electrode surface, promoting detachment and hence eliminating the hysteresis time required for the current to penetrate the passive film and enhancing the discharge activity of Mg alloy electrodes.

Notably, the Na_2_SiO_3_ addition reduced the discharge potential, which was −1.93 V in the composite electrolyte without Na_2_SiO_3_ at 3 mA cm^−2^. In particular, when $C_{{\text{Na}}_{2}{\text{SiO}}_{3}}$ was 6 mM, the discharge voltage occurred at −3.21 V, corresponding to a negative shift of 1,380 mV. This facilitated the increase in the output voltage of a battery cell. However, the discharge potential shifted positively with $C_{{\text{Na}}_{2}{\text{SiO}}_{3}}$ > 6 mM. It is speculated that at high Na_2_SiO_3_ concentrations, the excess SiO_3_^2−^ ions may react with Mg^2+^ ions in the electrolyte to form deposits on the electrode surface (Ge et al., 2013), potentially thickening the passive film on the electrode surface and hindering the discharge of the electrode, thus shifting the discharge potential to the positive direction.

**FIGURE 3:**
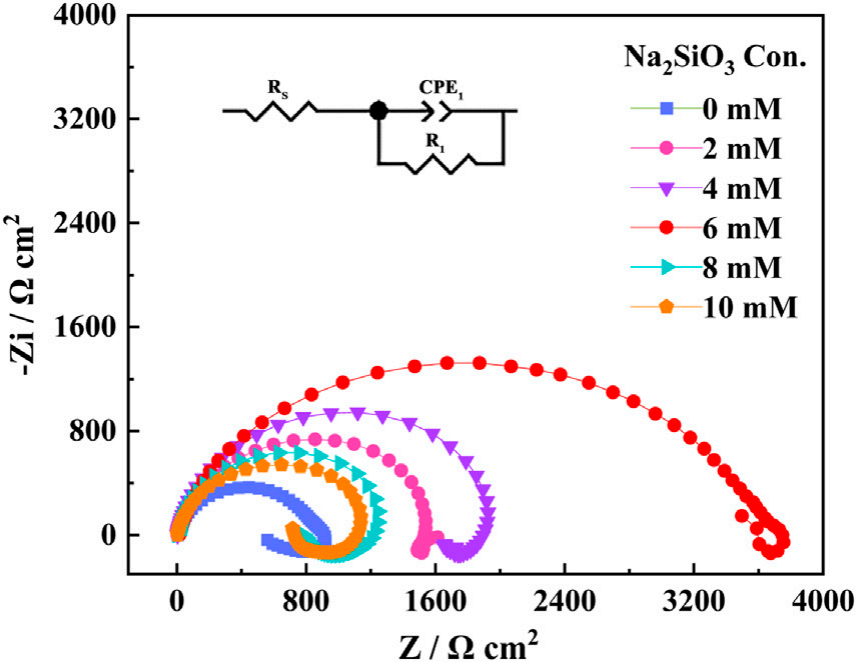
Nyquist curves of EIS for AZ31B Mg alloy electrodes in the Na_2_SO_4_-NaNO_3_ composite electrolyte with different concentrations of Na_2_SiO_3_.

The EIS spectra of the AZ31B Mg alloy electrodes after 24 h immersion in the composite electrolytes of varying Na_2_SiO_3_ concentrations are shown in Figure 3. EIS was conducted at an open-circuit potential of 10^–2^∼10^5^ Hz, with a sinusoidal disturbance amplitude of 5 mV. The insert illustrated the equivalent circuit of the EIS system. In the equivalent circuit diagram, *R*_s_ was induced by the solution resistance; 下标 corresponded to the high-frequency charge-transfer resistance arising from the alloy surface and corrosive film layer. *CPE*_1_ denoted the double-layer capacitance induced by irregular oxide film on the alloy surface.

As shown in Figure 3, the EIS plots exhibited capacitive loops of similar shapes at low and high frequencies, and only some changes took place in the radii of the capacitive loops, indicating that the corrosion mechanism of the AZ31B Mg alloy was free from the influence of the addition amount of Na_2_SiO_3_. At a Na_2_SiO_3_ concentration of 6 mM, the capacitive reactance diameter reached the maximum. According to the curves, *R*_1_ was 957 Ω in the absence of Na_2_SiO_3_. With the Na_2_SiO_3_ addition, the resistance value was enhanced significantly, indicating the prohibited corrosion of the AZ31B Mg alloy in the Na_2_SO_4_-NaNO_3_ composite electrolyte. When $C_{{\text{Na}}_{2}{\text{SiO}}_{3}}$ was 6 mM, the *R*_1_ value reached the maximum of 3,886 Ω, increasing by more than four times and representing a stronger corrosion resistance.

**FIGURE 4:**
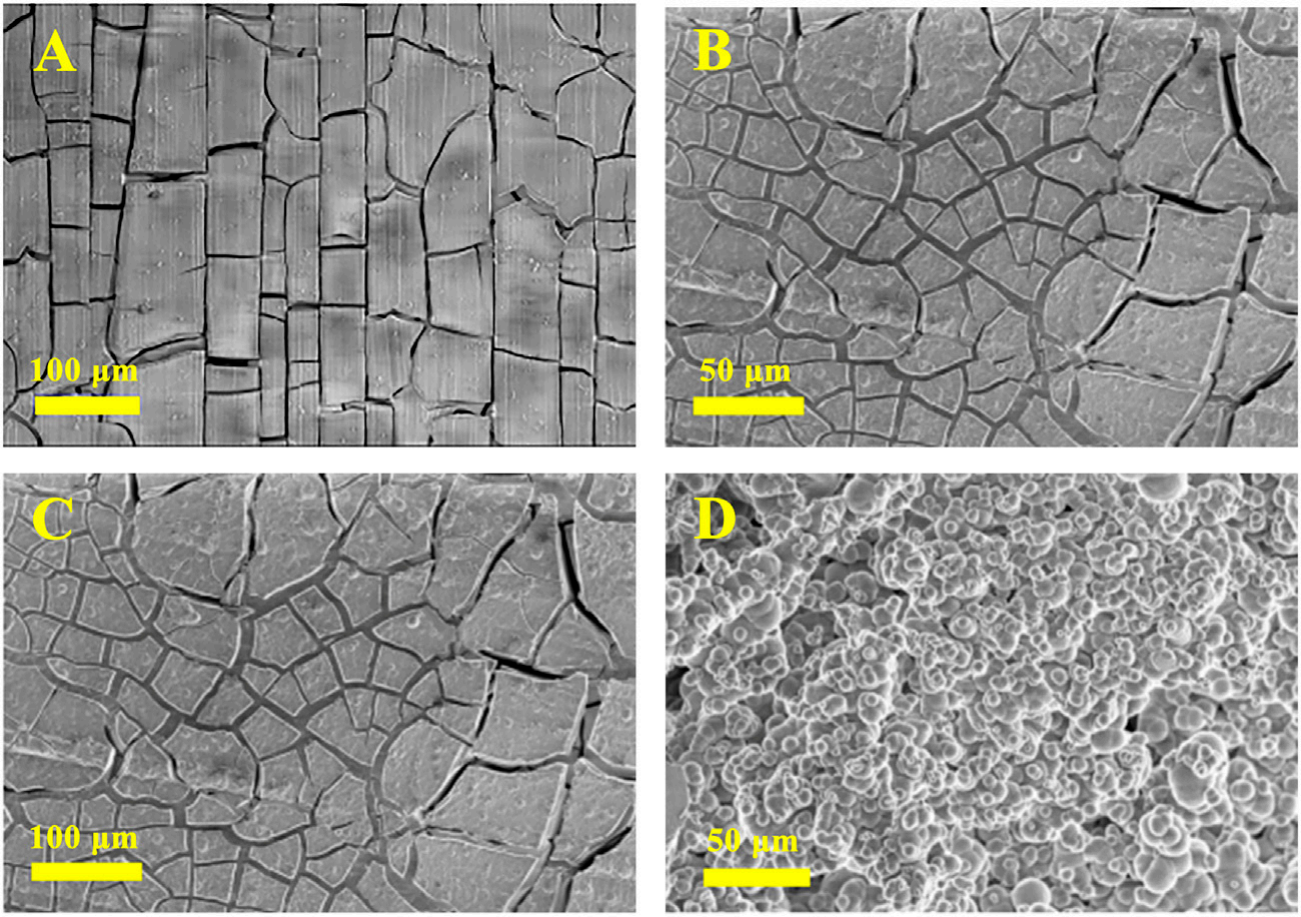
SEM of the AZ31B Mg alloy soaked in the Na_2_SO_4_-NaNO_3_ composite electrolyte in the absence **(A,B)** and presence **(C,D)** of Na_2_SiO_3_ for 24 h.

The surface morphology images of AZ31B Mg alloy electrodes after 24 h immersion in the Na_2_SO_4_-NaNO_3_ composite electrolyte are shown in Figure 4. Figures 4A,B show the SEM images of electrodes soaked in the absence of Na_2_SiO_3_. After 24 h of soaking, a thick, dense, and smooth film was formed on the Mg alloy electrode. Despite their large number, the cracks appearing on the surface were very narrow (generally <3 μm in width). As a result of hydrogen evolution from the electrode during the early soaking stages, a few small holes and pits were formed on the film. After 24 h of soaking in the presence of 6 mM Na_2_SiO_3_ (Figures 4C,D), the alloy surface exhibited more and wider cracks (the widest cracks exceeded 10 μm in width). A thick and dense film was formed, effectively protecting the Mg alloy electrode and enhancing its corrosion resistance.

## Conclusion

The influence of Na_2_SiO_3_ on the electrochemical and corrosion behavior of the AZ31B Mg alloy electrode in the Na_2_SO_4_-NaNO_3_ composite electrolyte (the volume ratio of 2 M Na_2_SO_4_ to 2 M NaNO_3_ was 1:9) was investigated in this work. When $C_{{\text{Na}}_{2}{\text{SiO}}_{3}}$ was 6 mM, *E*_act_ values reached −1.51 V, 1.1 V lower than that without the Na_2_SiO_3_ additive. In particular, the discharge voltage occurred at −3.21 V, shifting negative to 957 mV, and the discharge curves were rapidly stabilized. Moreover, the resistance value reached a maximum value of 3,886 Ω, increasing by more than four times. The composite electrolyte with 6 mM Na_2_SiO_3_ was appropriate for Mg alloy, loosening the passive film on the electrode surface, facilitating the ionic conductivity, and eliminating the hysteresis time. This can realize not only the excellent discharge activity but also the high corrosion resistance of Mg alloy electrodes. Therefore, the present work offers a new electrolyte formulation to enhance the electrochemical behavior and lifespan of Mg batteries.

## Data Availability

The raw data supporting the conclusions of this article will be made available by the authors without undue reservation.

## References

[B1] Bertasi F. Sepehr F. Pagot G. Paddison S. J. Di Noto V. (2016). Magnesium batteries: Toward a magnesium-iodine battery (adv. Funct. Mater. 27/2016). Adv. Funct. Mat. 27, 4859. 10.1002/adfm.201670174

[B2] Deng M. Wang L. Höche D. Lamaka S. V. Snihirova D. Vaghefinazari B. (2019). Clarifying the decisive factors for utilization efficiency of Mg anodes for primary aqueous batteries. J. Power Sources 441, 227201. 10.1016/j.jpowsour.2019.227201

[B3] Ge Y. F. Jiang B. L. Shi H. Y. (2013). Effect of Na_2_SiO_3_ concentration on energy consumption during arcing process of micro-arc oxidation on aluminum alloys. Chin. J. Nonferrous Mater. 23, 950–956. 10.19476/j.ysxb.1004.0609.2013.04.009

[B4] Gong C. Yan X. He X. Su Q. Liu B. Chen F. (2022). Influence of homogenization treatment on corrosion behavior and discharge performance of the Mg-2Zn-1Ca anodes for primary Mg-air batteries. Mat. Chem. Phys. 280, 125802. 10.1016/j.matchemphys.2022.125802

[B5] Horia R. Nguyen D. T. Eng A. Y. S. Seh Z. W. (2022). Comparative study of conventional electrolytes for rechargeable magnesium batteries. Batter. Supercaps 5, e202200011. 10.1002/batt.202200011

[B6] Kékedy-Nagy L. Abolhassani M. Greenlee L. F. Pollet B. G. (2021). An electrochemical study of ammonium dihydrogen phosphate on Mg and Mg alloy electrodes. Electrocatalysis 12, 251–263. 10.1007/s12678-021-00646-x

[B7] Kong D. Ren W. Qi L. Zhang Y. Chen H. (2022). Enhanced bonding strength of AZ31B/carbon-fiber-reinforced plastic laminates by anodization treatment in a saturated Na_2_SiO_3_ solution. Mater. Sci. Eng. A 840, 142982. 10.1016/j.msea.2022.142982

[B8] Li J. Wei Z. Gao Z. (2021). Improving corrosion and discharge performance of magnesium alloy via sodium stannate additive. J. Xinyang Normal Univ. Nat. Sci. Ed. 1, 93–98. 10.3969/j.issn.1003-0972.2021.01.015

[B9] Liu S. Li X. Z. Huang B. Yang J. W. Chen Q. Q. Li Y. W. (2021). Controllable construction of yolk-shell Sn-Co@void@C and its advantages in Na-ion storage. Rare Met. 40, 2392–2401. 10.1007/s12598-021-01729-w

[B10] Maddegalla A. Mukherjee A. Blázquez J. A. Azaceta E. Leonet O. Mainar A. R. (2021). Cover feature: AZ31 magnesium alloy foils as thin anodes for rechargeable magnesium batteries (ChemSusChem 21/2021). ChemSusChem 14, 4611. 10.1002/cssc.202102057 PMC8596635 34339584

[B11] Qu J. Luo H. Liu Z. Wang H. Chen Y. Yang L. (2022). Effect of sodium-zinc EDTA and sodium gluconate as electrolyte additives on corrosion and discharge behavior of Mg as anode for air battery. Mat. Corros. 2022, 1–12. 10.1002/maco.202213322

[B12] Shao Y. Rajput N. N. Hu J. Hu M. Liu T. Wei Z. (2015). Nanocomposite polymer electrolyte for rechargeable magnesium batteries. Nano Energy 12, 750–759. 10.1016/j.nanoen.2014.12.028

[B13] Wan M. Zeng R. Meng J. Cheng Z. Chen W. Peng J. (2022). Post-synthetic and *in situ* vacancy repairing of iron hexacyanoferrate toward highly stable cathodes for sodium-ion batteries. Nano-Micro Lett. 14, 9. 10.1007/s40820-021-00742-z PMC8642478 34862572

[B14] Wang N. Wang R. Peng C. Peng B. Feng Y. Hu C. (2014). Discharge and corrosion performance of AP65 magnesium alloy in simulated seawater: Effect of temperature. J. Mat. Eng. Perform. 23, 4374–4384. 10.1007/s11665-014-1222-2

[B15] Wei Q. Li Q. Jiang Y. Zhao Y. Tan S. Dong J. (2021). High-energy and high-power pseudocapacitor-battery hybrid sodium-ion capacitor with Na^+^ intercalation pseudocapacitance anode. Nano-Micro Lett. 13, 55. 10.1007/s40820-020-00567-2 PMC8187546 34138220

[B16] Wei Z. Pan Q. Han X. (2022). Effect of changes in geothermal water temperature on corrosion behavior of stainless steel pipes. J. Xinyang Normal Univ. Nat. Sci. Ed. 1, 113–117. 10.3969/j.issn.1003-0972.2022.01.019

[B17] Xu J. Yang Q. Huang C. Javed M. S. Aslam M. K. Chen C. (2017). Influence of additives fluoride and phosphate on the electrochemical performance of Mg-MnO_2_ battery. J. Appl. Electrochem. 47, 767–775. 10.1007/s10800-017-1074-1

[B18] Yang J. Miao X. Zhang C. Zheng J. Sun C. Zhang Y. (2022). *In-situ* lattice tunnel intercalation of vanadium pentoxide for improving long-term performance of rechargeable magnesium batteries. ChemNanoMat 8, e202200025. 10.1002/cnma.202200025

[B19] Zhang C. Wang A. Guo L. Yi J. Luo J. (2022). A moisture-assisted rechargeable Mg-CO_2_ battery. Angew. Chem. Int. Ed. Engl. 61, e202200181. 10.1002/anie.202200181 35170161

[B20] Zhang J. L. Li C. L. Wang W. H. Yu D. Y. W. (2021). Facile synthesis of hollow Cu_3_P for sodium-ion batteries anode. Rare Met. 40, 3460–3465. 10.1007/s12598-021-01718-z

[B21] Zhang Y. Zhao T. Li S. (2021). Influence of different morphologies on the supercapacitive performance of NiCo_2_O_4_ particles. J. Xinyang Normal Univ. Nat. Sci. Ed. 01, 99–104.

[B22] Zhao Y. C. Huang G. S. Gong G. L. Han T. Z. Xia D. B. Pan F. S. (2016). Improving the intermittent discharge performance of Mg-Air battery by using oxyanion corrosion inhibitor as electrolyte additive. Acta Metall. Sin. Engl. Lett.) 29, 1019–1024. 10.1007/s40195-016-0471-5

